# The Expression of Selected Proapoptotic Molecules in Dermatitis Herpetiformis

**DOI:** 10.1155/2012/178340

**Published:** 2012-06-20

**Authors:** Zebrowska Agnieszka, Erkiert-Polguj Anna, Wagrowska-Danilewicz Malgorzata, Danilewicz Marian, Sysa-Jedrzejowska Anna, Anna Cynkier, Waszczykowska Elzbieta

**Affiliations:** ^1^Department of Dermatology and Venereology, Medical University of Lodz, Krzemieniecka Street no. 5, 94-017 Lodz, Poland; ^2^Laboratory of Nephropathology, Medical University of Lodz, Pomorska street no. 251, 92-216 Lodz, Poland

## Abstract

The role of the process of apoptosis is investigated in the pathogenesis of many autoimmune diseases; however at present, there is not much information about its role in dermatitis herpetiformis. Skin biopsies were taken from 18 DH patients and from 10 healthy subjects. The localization and expression of Bax, Fas, FasL, TRAIL, TRAIL-R in skin lesions, and perilesional skin were studied by immunohistochemistry. Expression of Bax, Fas, and Fas ligand was detected in the keratinocytes in skin biopsies from DH patients. Expression of TRAIL and TRAIL receptor was confirmed in epidermis, infiltration cells, and some fibroblasts. The expression of examined molecules in biopsies from healthy people was observed only in single cells. There were statistically significant differences between lesional, perilesional, and healthy skin of control group in Bax expression analysis and between lesional skin and control group in Fas, FasL, and TRAIL expression. There were statistically significant differences between control group and perilesional skin in Bax and FasL expression. Our results show that selected proapoptotic molecules may take part in pathogenesis of dermatitis herpetiformis, but the role of apoptosis in this process is not clear.

## 1. Introduction

Dermatitis herpetiformis (DH) is a chronic subepidermal autoimmune bullous diseases characterized by skin and intestinal lesions. Skin lesions include polymorphic eruption (papules, vesicles), mainly distributed over the shoulders, elbows, backs, buttocks, and knees. They are usually symmetric and accompanied by severe pruritus. These symptoms are usually associated with asymptomatic and gluten-sensitive enteropathy. Diagnosis of DH is established on the results of direct immunofluorescence test (DIF) revealing granular deposits of IgA in the top of the papillae and the presence of circulating IgA antibodies directed against endomysium and/or tissue, and epidermal transglutaminase (tTG, eTG). Skin lesions in DH are histologically characterized by neutrophilic infiltrate leading to destruction of basement membrane zone (BMZ) proteins. Impairment of type IV collagen, laminin, and entactin results in degradation of anchoring fibres and blister formation. [1, 2] To date, the exact mechanisms that lead to lesions formation, are only partially known.

The role of the process of apoptosis is investigated in the pathogenesis of many autoimmune diseases; however at present, there is not much information about its role in subepidermal blistering diseases.

Apoptosis, also known as programmed death of a cell, is an outcome of the intracellular cell “suicide,” which is regulated by cellular pathways of passing the signal. It prevents many pathological processes, for example, autoimmunization and neoplasm [3, 4].

Up to this day, two pathways of apoptosis have been described, that is, intrinsic and extrinsic and the basis for its distinction is a way of activation of the procaspases which initiate them [5, 6].

The intrinsic one, called mitochondrial, is connected with the activation of cytochrome c by the proapoptotic genes belonging to Bcl-2 family (*B-cell leukemia/lymphoma-2*) as a result of, for example, medicine administered or the destruction of DNA structure [7]. The features triggering the mitochondrial pathway of death can be for example: the increase in concentration of the reactive forms of oxygen, nitrogen oxide, ions Ca^2+^, thermal shock, active toxins, the disturbance of the electron transport, or DNA damage [6].

The extrinsic pathway is associated with attaching the ligands to the receptor belonging to the superfamily of TNF receptors, which possess the so-called *death domain* (DD), by means of which the activation of the procaspases inside the cell occurs [3].

The intrinsic and extrinsic pathways stimulating apoptosis are linked to each other by, for example, Bid protein, belonging to Bcl-2 family [6].

The proteins composing the superfamily of TNF receptor also take an active part in the process of apoptosis. These are Fas, TRAIL-R1 (DR4), TRAIL-R2 (DR5, Apo2), TNF-R1, TNF-R2, TRAMP (*TNF-related apoptosis-mediating protein*), and DR-6 [8]. The pathway of operation of the Fas ligand on the receptor Fas has been most thoroughly described. At present, it is believed that the constitutive coexpression of the receptor and the ligand Fas takes place in the cells of the rapid apoptotic turnover [9].

As TNF-R, Fas, and TRAIL receptors appear on the keratinocytes, they can take part in the pathogenesis of some skin diseases such as: toxic epidermal necrolysis, Graft-versus-host disease, skin neoplasms, and contact oversensitivity [8].

In the tissue material *ex vivo*, the apoptosis is difficult to determine quantitatively because of the dynamics of the process; therefore, the number of the registered apoptotic cells often constitute only a small percentage of the total number of cells which entered the state of apoptosis [10]. In general, the majority of cells of the hematopoietic line atrophies in the process of apoptosis and manifests the typical features of apoptosis, while the death of the epithelial cells is more complicated and very often hard to classify [11].

## 2. Material and Methods

The study of selected proapoptotic proteins (Bax, TRAIL, TRAIL-R-DR4, Fas, Fas ligand) was performed on 18 patients (age: 44.8 years; range: 18–58 years) with dermatitis herpetiformis, who were treated in Department of Dermatology and Venereology of Medical University of Lodz. The patients were before treatment, at an active stage of the disease, that is, with skin lesions (erythemas, papules, vesicles) developed. The lesions were accompanied by itch of different intensification. DH was diagnosed based on clinical picture, histological, and immunological findings.

10 healthy volunteers, selected according to their sex and age, made up the control group.

All the participants of the experiment gave an explicit consent in writing before entering the study and the study protocol was approved by The Local Ethical Committee of Medical University of Lodz (no. RNN/132/07/KE, 20.02.2007).

The biopsies from all patients were taken from lesional and from uninvolved skin (trunk) before administration of any treatment (topical or systemic). In control group, biopsy specimens were taken from buttock or abdominal skin of healthy volunteers.

Paraffin-embedded sections (3-4 *μ*m) were used for routine H + E staining and for immunohistochemistry in DAKO EnVision detection system using immunoperoxidase method. The following primary monoclonal antibodies were used: Bax (Dako, Denmark) (1 : 650), TRAIL (Abcam, the United States) (1 : 200), TRAIL-R (R&D, the United Kingdom) (1 : 200), Fas, Fas ligand (Novocastra, the United Kingdom) (1 : 200).

For immunohistochemistry, the paraffin-embedded sections were placed on adhesive plates and dried at 56°C for 24 hours. Later, they were deparaffinated in a series of xylenes and alcohols with decreasing concentrations (96%, 80%, 70%, and 60%). In order to retrieve the antigenicity of tissues and allow them to react with antibodies, sections were prepared in bath (98°C-1 h) or microwave oven. Then the sections were washed with TRIS buffer (pH 7.6) for 5 min. Activity of endogenous peroxidase was inhibited with 0.3% hydrogen peroxide solution in methanol for 30 minutes. Primary antibody solution directed against human antigens was put on these sections. After incubation with diluted antibodies for 60 minutes at room temperature or for 12 hours at 4°C, they were washed with TRIS buffer twice. DAKO EnVision double-step visualization system was then used in order to visualize the antigen-antibody reaction. In cases of positive immunohistochemical reaction, cellular nuclei were stained with Meyer haematoxylin for 2 minutes. After dehydratation and processing through series of acetones and xylenes, the sections were fixed in DPX. For every antibody a negative control was performed using TRIS buffer instead of antibody.

### 2.1. Semiquantitative Analysis

In each specimen staining intensity of TRAIL and TRAIL receptor, Fas as well as Fas ligand in the epithelium and inflammatory infiltrates were recorded by two independent observers in 4–7 adjacent high power fields and graded from 0 (staining not detectable), 1 (minimal immunostaining in some cells), 2 (weak immunostaining intensity in most cells), and 3 (strong staining in most cells). The mean grade was calculated by averaging grades assigned by the two authors and approximating the arithmetical mean to the nearest unity.

Morphometric analysis was used for Bax (MultiScan 8.08 software, Computer Scanning System, Polska). The percentage of Bax immunopositive cells was estimated by counting in each slide 500 cells in 4–7 adjacent high power fields (semiautomatic function).

All data are shown as mean ± SD. Student's *t*-test was applied where appropriate after evaluation of distribution. Mann-Whitney test was used where necessary. The difference was considered statistically significant when *P* < 0.05.

## 3. Results

**Figure 1:**
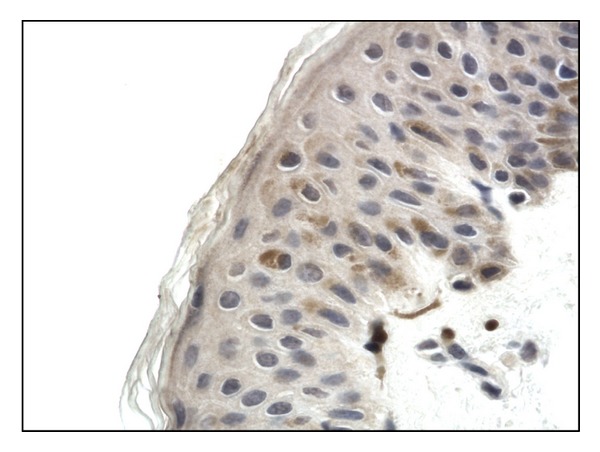
Cytoplasmic Bax immunoexpression in skin lesions, (score 2), 400x.

Bax protein expression was discovered in the cytoplasm of keratinocytes in samples of lesional skin (mean immunoexpression 39.742 ± 7.295) (Figure 1). The expression was weaker in uninvolved skin (22.347 ± 3.814) and the weakest Bax expression was revealed in samples taken from healthy volunteers (16.60 ± 3.6).

**Figure 2:**
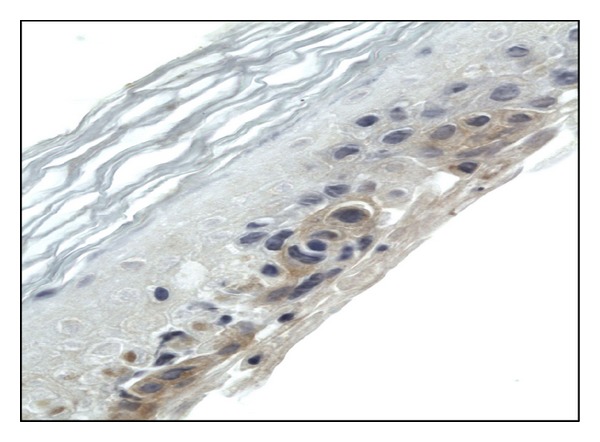
Fas immunoexpression in the cytoplasm of keratinocytes, (score 2), 400x.

Fas expression in lesional skin was detected in the cytoplasm of keratinocytes (0.75 ± 0.347) (Figure 2). In uninvolved skin, the expression was less intense 0.306 ± 0.290. None of the samples taken from healthy patients revealed Fas expression.

**Figure 3:**
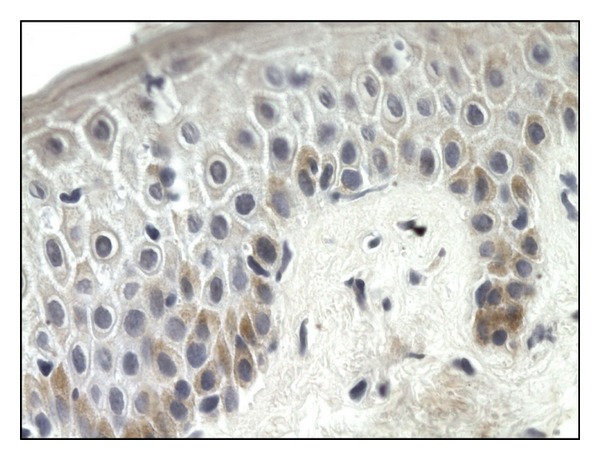
Fas ligand immunoexpression in the basal layer of the epidermis, (score 1), 400x.

Fas ligand expression was revealed in the basal layer of the epidermis and inflammatory infiltrates in lesional skin (0.923 ± 0.427) (Figure 3). In uninvolved skin, the expression was weaker in the basal layer of the epidermis and in few cells infiltrating the skin (0.66 ± 0.292). Immunostaining for Fas ligand was negative in control group.

**Figure 4:**
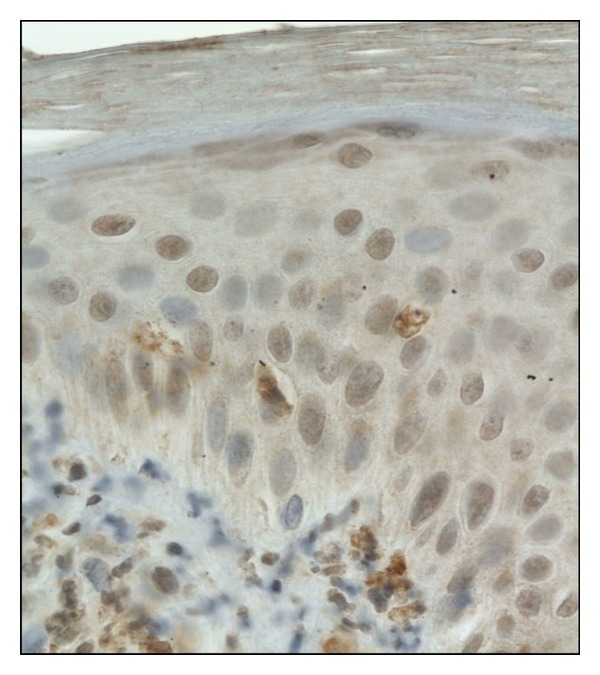
TRAIL immunoexpression in the cytoplasm of keratinocytes and inflammatory cells, (score 1), 400x.

Immunostaining of TRAIL was detected in the cytoplasm of keratinocytes as well as in inflammatory cells, and some fibroblasts in lesional skin (1.555 ± 0.783) (Figure 4). In uninvolved skin, the expression was detected in keratinocyte, mainly of the basal layer, and in some fibroblasts was less intense (0.468 ± 0.304). As for healthy skin, the expression was discovered in keratinocytes and in some fibroblasts (0.25 ± 0.191).

**Figure 5:**
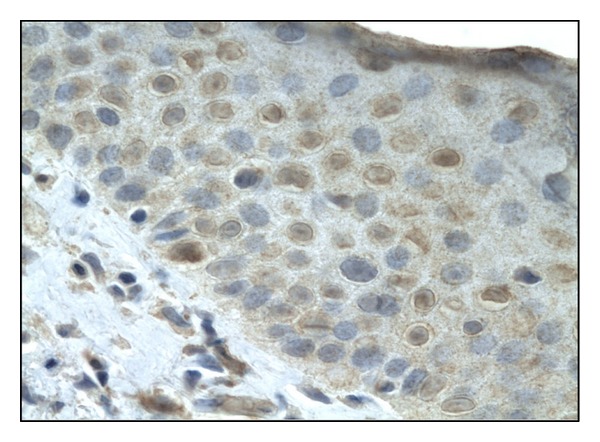
TRAIL receptor immunoexpression in skin lesions, (score 2), 400x.

Immunostaining of TRAIL receptor was revealed in inflammatory infiltration, in the cytoplasm of keratinocytes as well as in some fibroblasts in lesional skin (0.735 ± 0.634) (Figure 5), in uninvolved skin (1.005 ± 0.639), and in healthy skin (0.5 ± 0.258).

There were statistically significant differences between lesional, perilesional, and healthy skin of control group in Bax expression analysis. The expression of Fas and TRAIL was higher in lesional skin than in perilesional, and in healthy group. FasL expression was significantly higher in skin lesions and perilesional skin than in control group. In TRAIL-R analysis, there was no statistically significant difference, although the expression of TRAIL-R was more intense in uninvolved than in lesional skin.

Statistical analysis was presented in Table 1.

## 4. Discussion

Apoptosis is claimed to be involved in a number of chronic inflammatory and neoplastic skin diseases such as contact dermatitis, toxic epidermal necrolysis, acantholytic dermatoses, and systemic lupus erythematosus [3, 4, 12, 13], also may play role in pathogenesis of bullous diseases, although there are only a few works considering apoptosis in DH. Mikalowski [14] showed cytolysis and desmosomal alterations in basal keratinocytes in DH skin lesions, that is a sign of cellular damage.

Caproni et al. [15] examined apoptosis in 13 DH patients by TUNEL method (terminal deoxynucleotidyl transferase-mediated deoxyuridine triphosphate nick end labelling technique). Apoptosis was seen in the basal and suprabasal layers of epidermis. In dermis, in the area of blood vessels, there were only a few apoptotic cells. In skin biopsies taken from the healthy control group there were no apoptotic cells.

Proapoptotic Bcl-2 family consists of many proteins for example, Bax, that takes part in intracellular pathway of apoptosis. In nonapoptotic cells, Bax and Bcl-2 form heterodimers keeping the homeostasis [6]. In our study, Bax expression was detected in keratinocytes of suprabasal layers, which was observed in earlier studies [16].

Caproni et al. [15] also examined the expression of proapoptotic protein Bax and antiapoptotic Bcl-2 in lesional skin. In our study similar to Caproni et al., Bax expression was seen in basal layer of epidermis and in papillary dermis and was more intense in lesional than perilesional skin. Bcl-2 expression was present in epidermis and in dermis near superficial vessels.

The results of our study showed significant difference of Bax expression between lesional, perilesional, and healthy skin; that is why the overexpression of Bax protein seems to take part in DH pathogenesis, but explanation of the precise mechanism of starting the intrinsic pathway of apoptosis needs more studies.

Extrinsic pathway of apoptosis starts for example, after activation of Fas receptor (also known as APO-1 or CD95) by its ligand FasL [4]. In physiological condition, Fas expression is weak and is present in cell membrane, or in intracellular compartment, which prevents spontaneous apoptosis [17–19]. Although in physiological condition FasL is present on keratinocytes in granular layer, spinous layer and external hair shaft however, neither we, nor Caproni et al., [15] detected Fas and FasL expression in healthy skin from control group.

Also in other bullous diseases, Fas was examined, Morawej et al. [20] described the elevation of soluble Fas (sFas) in pemphigus vulgaris patients' sera and suggest that particularly in the initial phases of autoimmune disorders, sFas may take part in the resistance of autoreactive lymphocytes to death.

Also our earlier research [21], considering pemphigoid, showed overexpression of Fas and FasL in lesional skin. In this DH study, we observed expression of Fas and FasL in epidermal cells, and FasL was seen also in inflammatory infiltration. Studies of other authors showed FasL on activated T cells, neutrophils, natural killer cells, and macrophage lineage other cell types. [4, 22, 23] In DH neutrophils and T cells are main cells of inflammatory infiltration, therefore, we suggest that infiltrating cells with FasL on the surface act on keratinocytes with Fas receptor. The connection of ligand to Fas receptor triggers intracellular cascade leading to apoptosis. Expression of both Fas on keratinocytes and FasL cells in the same topographic place implies that after the ligand and receptor have joined apoptosis gets started.

Similar data showed Caproni et al. [15] described Fas expression on basal keratinocytes in lesional and perilesional skin in DH and same in perilesional skin within perivascular superficial dermis. Higher expression of epidermal Fas than FasL and inversely in dermis, in the authors' opinion, indicates importance of extrinsic pathway of apoptosis in skin lesion formations [15].

 Fas has not only proapoptotic activity, but also proinflammatory one [24, 25]. Inflammatory activity of Fas showed Farley et al. [24], who described the influence of FasL on Fas receptor which leads to its oligomerization without caspase activation. The effect of that process is not apoptosis, but expression of genes of proinflammatory factors, for example, IL-6, IL-8, MCP-1. This way of activation cannot be excluded in DH, because overexpression of many proinflammatory cytokines (e.g., GM-CSF, IL-4, IL-5, IL-8 [26, 27] was observed in this disease. Although, in our earlier studies MCP-1 was absent in DH lesional and perilesional skin, but we reported receptors for IL-8 [28].

Many factors, such as UV radiation, cytokines (e.g., IL-1*β*, TNF-*α*, IFN-*γ*, and IL-15), viral infections (hepatitis B or C, HIV infection) can induce expression of Fas in epidermis [4, 29]. More precise studies are needed to establish which of these factors can take part in DH.

Other proapoptotic protein of extracellular way of apoptosis activation is TRAIL, also known as Apo-2L. TRAIL-R1 (DR4) and TRAIL-R2 (DR5), which transduces the death signal and induces apoptotic cell death [30, 31]. Although both of them coexist on keratinocytes, DR4 seems to be more effective [32]. These receptors do not take part in terminal differentiation of keratinocytes [33]. There are also TRAIL receptors on keratinocytes which do not promote apoptosis, because recombinant TRAIL does not induce apoptosis on healthy keratinocytes. By decoy receptors, TRAIL activates NF-*κ*B, which leads to transcription of genes that are antagonists of caspase 8 and promote inflammatory process. In endothelial cells line which avoids TRAIL-induced apoptosis, expression of E-selectin, ICAM-1, IL-8, and IL-1Ra is observed. That leads to lymphocyte adhesion [5, 32, 34]. In our earlier study [28, 35] thus, we observed overexpression of E-selectin and receptors for IL-8 in dermatitis herpetiformis.

 The role of TRAIL in physiological condition is not completely known. *In vitro* TRAIL is a stronger proapoptotic factor than TNF. In spite of that, it seems that most of cells are resistant to TRAIL-induced apoptosis. TRAIL protein is constitutively present on many cells, but in T and NK cells and dendritic blood cells, it is synthesized after their stimulation [13]. Interferon stimulation on neutrophils makes the expression of TRAIL is detected. mRNA for DR2 and DR4-TRAIL receptors constitutively is found in neutrophils [36].

The role of TRAIL in autoimmune diseases is discussed. Some researchers suggest that high level of TRAIL can increase disease activity, whereas some others present opposite opinion. TRAIL can induce the process of apoptosis of dendritic cells and neutrophils and its high expression was seen in T cells infiltrating skin in atopic dermatitis [13]. In our study, TRAIL and its receptor DR4 expression was present on keratinocytes and infiltarting cells. The TRAIL expression was significantly higher in lesional than perilesional and control group skin, which may suggest its role in DH pathogenesis. The influence of TRAIL on human eosinophils did not induce chemotactic effect, but led to apoptosis [36], therefore, the presence of TRAIL and its receptor in infiltrating cells seems to be related to apoptosis.

The recent study of Wu et al. [37] reported that TRAIL can induce the expression of the keratinocyte differentiation markers involucrin and type 1 transglutaminase in normal human epidermal keratinocytes. Activation of caspases 3 and 8 critically mediates these processes, but apoptosis can also be triggered.

Higher expression of TRAIL receptor in perilesional rather than lesional skin seems interesting, but these results were not statistically significant. The more intense in uninvolved than in lesional skin expression of TRAIL receptor DR4 on keratinocytes and cells of inflammatory infiltration may suggest its role in maintaining inflammatory process and damage to dermoepidermal junction.

The proteins from intrinsic (Bax) and extrinsic (Fas and TRAIL) pathway of apoptosis activation were assessed in our study. However, some authors hold the opinion that the recognition of autonomy of these two apoptotic ways is a simplification, as there are proteins which connect these two ways, for example, Bid protein [6]. Also Suliman et al. [38] showed that TRAIL besides activation of extrinsic way, decreases transmembrane potential in mitochondria and induces intrinsic way of apoptosis.

 Caproni et al. [15] state that apoptosis in DH seems to be induced by extrinsic pathway, but other factors can also be influential: hypoxia due to mechanical tension of blister fluid or loss of link between the skin and epidermis may enhance the apoptotic process.

Our earlier study [21] considered the same molecules in bullous pemphigoid (BP). The Bax, Fas, FasL, TRAIL, and TRAIL-R were overexpressed in lesional skin in comparison to healthy control. Although the semiquantitative visual scale and morphometric analysis showed that immunostaining of examined proteins was more intense in DH comparing to BP. However, in Caproni et al. [15] study, the epidermal expression of Bax in BP was significantly higher than in DH. The difference may be the result of number of examined biopsies (BP: 5 [15] versus 22 [21], DH: 13 [15] versus 18 [21]). Next, also *in vitro* research seems to be needed to clarify the role of apoptosis in DH.

Our results as well as studies conducted by other authors showed that the role of apoptosis in DH pathogenesis is not clear. The exact stimulus triggering apoptosis in DH skin lesion still remains unknown, but the present study provides strong evidence that there is a difference between expression proapoptotic proteins in skin lesions, perilesional skin, and healthy skin. Cell death by apoptosis is a physiological process that enables the elimination of cells without causing an inflammatory response, but inflammation can provoke apoptosis so this mechanism in DH should be more explained.

## Figures and Tables

**Table 1:** Expression of proteins in examined tissues. Statistical results. Data expressed as means ± SD; ^∗^ *P* < 0.05.

Groups	Protein				
	BAX means ± SD	Fas means ± SD	FasL means ± SD	TRAIL means ± SD	TRAIL-R means ± SD
DH skin lesions	39.742 ± 7.295	0.75 ± 0.347	0.923 ± 0.427	1.555 ± 0.783	0.735 ± 0.634
DH perilesional skin	22.347 ± 3.814	0.3060 ± 0.29	0.66 ± 0.292	0.468 ± 0.304	1.005 ± 0.639
Control group	16.60 ± 3.6	0.0	0.0	0.25 ± 0.191	0.5 ± 0.258
Control versus DH skin lesions	*P* < 0.001	*P* < 0.001	*P* < 0.001	*P* < 0.004	*P* = 0.4 (NS)
Control versus DH perilesional skin	*P* < 0.02	*P* = 0.06 (NS)	*P* < 0.001	*P* = 0.18 (NS)	*P* = 0.15 (NS)
DH skin lesions versus DH perilesional skin	*P* < 0.001	*P* < 0.001	*P* = 0.06 (NS)	*P* < 0.001	*P* = 0.19 (NS)

## References

[1] Caproni M, Antiga E, Melani L, Fabbri P, Italian Group for Cutaneous Immunopathology (2009). Guidelines for the diagnosis and treatment of dermatitis herpetiformis. *Journal of the European Academy of Dermatology and Venereology*.

[2] Reunala TL (2001). Dermatitis herpetiformis. *Clinics in Dermatology*.

[3] Edinger AL, Thompson CB (2004). Death by design: apoptosis, necrosis and autophagy. *Current Opinion in Cell Biology*.

[4] Teraki Y, Shiohara T (1999). Apoptosis and the skin. *European Journal of Dermatology*.

[5] Kiliańska ZM, Miśkiewicz A (2003). Kaspazy kręgowców; ich rola w przebiegu apoptozy. *Posępyt Bioogii Komórki*.

[6] Reed JC (2000). Mechanisms of apoptosis. *The American Journal of Pathology*.

[7] Grądzka I (2000). Apoptoza: decyzja należy do mitochondrium. *Postępy Biochemii*.

[8] Wehrli P, Viard I, Bullani R, Tschopp J, French LE (2000). Death receptors in cutaneous biology and disease. *Journal of Investigative Dermatology*.

[9] French LE, Tschopp J (1996). Constitutive Fas ligand expression in several non-lymphoid mouse tissues: implications for immune-protection and cell turnover. *Behring Institute Mitteilungen*.

[10] Sulejczak D (2000). Apoptoza i metody jej identyfikacji. *Postępy Biologii Komórki*.

[11] Darzynkiewicz Z, Juan G, Li X, Gorczyca W, Murakami T, Traganos F (1997). Cytometry in cell necrobiology: analysis of apoptosis and accidental cell death (necrosis). *Cytometry*.

[12] Viard I, Wehrli P, Bullani R (1998). Inhibition of toxic epidermal necrolysis by blockade of CD95 with human intravenous immunoglobulin. *Science*.

[13] Vassina E, Leverkus M, Yousefi S, Braathen LR, Simon HU, Simon D (2005). Increased expression and a potential anti-inflammatory role of TRAIL in atopic dermatitis. *Journal of Investigative Dermatology*.

[14] Mikalowski R (1964). Microscopic electronique de l’epiderme dans les bulles de la dermatite herpetiforme de Dühring-Brocq. *Annales Dermatology Syphilology*.

[15] Caproni M, Torchia D, Antiga E (2005). The role of apoptosis in the pathogenesis of dermatitis herpetiformis. *International Journal of Immunopathology and Pharmacology*.

[16] Tomková H, Fujimoto W, Arata J (1998). Expression of the bcl-2 homologue bax in normal human skin, psoriasis vulgaris and non-melanoma skin cancers. *European Journal of Dermatology*.

[17] Viard-Leveugle I, Bullani RR, Meda P (2003). Intracellular localization of keratinocyte Fas ligand explains lack of cytolytic activity under physiological conditions. *The Journal of Biological Chemistry*.

[18] Oishi M, Maed’a K, Sugiyama S (1994). Distribution of apoptosis-mediating Fas antigen in human skin and effects of anti-Fas monoclonal antibody on human epidermal keratinocyte and squamous cell carcinoma cell lines. *Archives of Dermatological Research*.

[19] Lee SH, Jang JJ, Lee JY (1998). Fas ligand is expressed in normal skin and in some cutaneous malignancies. *British Journal of Dermatology*.

[20] Morawej H, Yousefi M, Farrokhi B, Mosaffa N (2011). Soluble Fas in pemphigus vulgaris. *Archives of Iranian Medicine*.

[21] Erkiert-Polguj A, Żebrowska A, Wągrowska-Danilewicz M, Danilewicz M, Nykiel A, Waszczykowska E (2011). Expression of selected pro-apoptotic proteins in pemphigoid. *Advances in Dermatology and Allergology*.

[22] Irmler M, Thome M, Hahne M (1997). Inhibition of death receptor signals by cellular FLIP. *Nature*.

[23] Glass A, Walsh CM, Lynch DH, Clark WR (1996). Regulation of the Fas lytic pathway in cloned CTL. *The Journal of Immunology*.

[24] Farley SM, Dotson AD, Purdy DE (2006). Fas ligand elicits a caspase-independent proinflammatory response in human keratinocytes: implications for dermatitis. *Journal of Investigative Dermatology*.

[25] Wajant H, Pfizenmaier K, Scheurich P (2003). Non-apoptotic Fas signaling. *Cytokine and Growth Factor Reviews*.

[26] Graeber M, Baker BS, Garioch JJ, Valdimarsson H, Leonard JN, Fry L (1993). The role of cytokines in the generation of skin lesions in dermatitis herpetiformis. *British Journal of Dermatology*.

[27] Caproni M, Feliciani C, Fuligni A (1998). Th2-like cytokine activity in dermatitis herpetiformis. *British Journal of Dermatology*.

[28] Zebrowska A, Erkiert-Polguj A, Wągrowska-Danilewicz M (2009). Eotaxin (CCL11), TARC [thymus and activation-regulated chemokine (CCL17)], MCP-1 (CCL2) and CCR-1, CXCR-1, CXCR-2 expression in dermatitis herpetiformis and bullous pemphigoid. *Archives of Medical Science*.

[29] Frisch SM, Francis H (1994). Disruption of epithelial cell-matrix interactions induces apoptosis. *Journal of Cell Biology*.

[30] Pan Y, Xu R, Peach M (2011). Evaluation of pharmacodynamic biomarkers in a phase 1a trial of dulanermin (rhApo2L/TRAIL) in patients with advanced tumours. *British Journal of Cancer*.

[31] Sheridan JP, Marsters SA, Pitti RM (1997). Control of TRAIL-induced apoptosis by a family of signaling and decoy receptors. *Science*.

[32] Leverkus M, Sprick MR, Wachter T (2003). TRAIL-induced apoptosis and gene induction in HaCaT keratinocytes: differential contribution of TRAIL receptors 1 and 2. *Journal of Investigative Dermatology*.

[33] De Panfilis G, Semenza D, Lavazza A, Mulder AA, Mommaas AM, Pasolini G (2002). Keratinocytes constitutively express the CD95 ligand molecule on the plasma membrane: an in situ immunoelectron microscopy study on ultracryosections of normal human skin. *British Journal of Dermatology*.

[34] Li JH, Kirkiles-Smith NC, McNiff JM, Pober JS (2003). Trail induces apoptosis and inflammatory gene expression in human endothelial cells. *The Journal of Immunology*.

[35] Zebrowska A, Waszczykowska E, Wągrowska-Danilewicz M (2009). Expression of selected adhesion molecules in dermatitis herpetiformis and bullous pemphigoid. *Polish Journal of Pathology*.

[36] Renshaw SA, Parmar JS, Singleton V (2003). Acceleration of human neutrophil apoptosis by TRAIL. *The Journal of Immunology*.

[37] Wu NL, Lee TA, Tsai TL, Lin WW (2011). TRAIL-induced keratinocyte differentiation requires caspase activation and p63 expression. *Journal of Investigative Dermatology*.

[38] Suliman A, Lam A, Datta R, Srivastava RK (2001). Intracellular mechanisms of TRAIL: apoptosis through mitochondrial-dependent and -independent pathways. *Oncogene*.

